# Treatment of severe fear of childbirth with haptotherapy: design of a multicenter randomized controlled trial

**DOI:** 10.1186/1472-6882-14-385

**Published:** 2014-10-08

**Authors:** Gert A Klabbers, Klaas Wijma, K Marieke Paarlberg, Wilco HM Emons, Ad JJM Vingerhoets

**Affiliations:** Therapy Center Ietje Kooistraweg 25, 7311 GZ Apeldoorn, the Netherlands; Unit of Medical Psychology, Department of Clinical and Experimental Medicine, Linköping University, SE 58183 Linköping, Sweden; Gelre Teaching Hospital Apeldoorn, Albert Schweitzerlaan 31, 7334 DZ Apeldoorn, the Netherlands; Department of Methodology and Statistics, Tilburg University, Warandelaan 2, 5037 AB Tilburg, the Netherlands; Department of Medical and Clinical Psychology Tilburg University, Warandelaan 2, 5037 AB Tilburg, the Netherlands

**Keywords:** Pregnant women, Fear of childbirth, Haptotherapy, Treatment, Delivery, Anxiety, Well-being, Childbirth outcomes

## Abstract

**Background:**

About six percent of pregnant women suffer from severe fear of childbirth. These women are at increased risk of obstetric labour and delivery interventions and pre- and postpartum complications, e.g., preterm delivery, emergency caesarean section, caesarean section at maternal request, severe postpartum fear of childbirth and trauma anxiety. During the last decade, there is increasing clinical evidence suggesting that haptotherapy might be an effective intervention to reduce fear of childbirth in pregnant women. The present study has been designed to evaluate the effects of such intervention.

**Methods/Design:**

Included are singleton pregnant women with severe fear of childbirth, age ≥ 18 year, randomised into three arms: (1) treatment with haptotherapy, (2) internet psycho-education or (3) care as usual. The main study outcome is fear of childbirth. Measurements are taken at baseline in gestation week 20–24, directly after the intervention is completed in gestation week 36, six weeks postpartum and six months postpartum. Secondary study outcomes are distress, general anxiety, depression, somatization, social support, mother-child bonding, pregnancy and delivery complications, traumatic anxiety symptoms, duration of delivery, birth weight, and care satisfaction.

**Discussion:**

The treatment, a standard haptotherapeutical treatment for pregnant women with severe fear of childbirth, implies teaching a combination of skills in eight one hour sessions. The internet group follows an eight-week internet course containing information about pregnancy and childbirth comparable to childbirth classes. The control group has care as usual according to the standards of the Royal Dutch Organisation of Midwives and the Dutch Organization of Obstetrics and Gynaecology.

**Trial registration:**

This trial was entered in the Dutch Trial Register and registered under number NTR3339 on March 4^th^, 2012.

## Background

Fear of childbirth (FOC) is a normally distributed phenomenon in the population of pregnant women. The level is slightly higher in primiparous women than in multiparous women [1]. A significant minority of pregnant women (6%) suffers from severe FOC, which negatively interferes with daily functioning [2, 3]. Zar et al. found that, according to DSM IV criteria [4], 2.4% of pregnant women suffer from phobic FOC [5], i.e. being so afraid of giving birth that they request a caesarean section in order to avoid vaginal birth. In the Netherlands, in 2012, 173.000 deliveries took place [6], meaning that annually about ten thousand women suffer from severe FOC. Until now, as far as we know, only two randomized clinical trials have been published on interventions treating severe FOC [7, 8]. In these studies positive effects have been reported of psycho-education in a group nulliparous women with FOC. In the most recent study, the intervention was associated with lower caesarean sections, more spontaneous vaginal deliveries and more satisfactory delivery experiences [8]. Haptotherapy (HT) has been applied in the clinical setting with promising results [9] but without scientific confirmation. Therefore, the present study has been specially designed to evaluate the effect of haptotherapy as a model for treatment of severe FOC.

### Characteristics of the women with fear of childbirth

Women with FOC are concerned about the well-being of themselves and of their infants [10, 11], the labour process (pain, medical interventions, abnormal course of labour, death, re-experiencing a previous traumatic delivery) [12], personal conditions (lack of control, distrust in own abilities) and external conditions (interaction with or the assistance of the staff) [13]. FOC is determined by the way a woman processes her sensations cognitively and emotionally. Her concerns about what may happen during an imminent or future delivery are crucial to this fear [14]. The event of childbirth is momentous for the woman giving birth, for the child being born, and for the woman’s partner. Her accomplishments during the delivery have lifelong physical, social and existential consequences for herself and her loved ones [1]. A woman with FOC has a propensity to worry about her ability to deal with possible obstetric problems, her capacity to perform adequately and the health, or even survival, of herself and her child during and after the delivery. Therefore, women with severe FOC not only continuously and apprehensively are vigilant for signals of danger, they often feel to have their suspicions verified [1], creating a vicious cycle of adverse expectations and negative experiences [1]. Some pregnant women with a strong inclination to worry about delivery, may even completely avoid childbirth information [1]. Towards the end of the pregnancy, some of these women may suddenly find themselves caught in a situation that they were not even able to contemplate. In such a situation, the women’s FOC may escalate to such high levels that their attention increasingly narrows and finally fully concentrates on fear-related stimuli [1].

### State-and trait-anxiety

Severe FOC may be considered from both a state and a trait perspective. For most pregnant women, the prospect of labour and delivery evokes a certain degree of uncertainty, perhaps even worry or fear. This may be called `state-anxiety` (situational FOC). The degree of state-anxiety during childbirth depends on how labour and delivery are progressing, the woman’s interpretation of what is happening, her propensity to view situations as hazardous or threatening and, finally, her ability to cope with what she perceives as difficult and dangerous [15, 16]. FOC as a `state` condition is a short-term reaction that waxes and wanes. FOC as a 'trait’ condition is, generally, more a characteristic of the woman, emphasizing her predisposition to react with fear to all kind of stimuli, including childbirth. FOC as a trait will influence fear levels both before and after the delivery. Individual differences in trait-anxiety may be the result of genetic factors as well as past experiences, such as the amount of negative information the expectant mother has received or collected about childbirth and her own life experiences. Women with high trait-anxiety levels show state-anxiety elevations more frequently than their low trait-anxiety counterparts [5]. Women with higher trait-anxiety further tend to regard a broader range of aspects of the delivery as dangerous or threatening [1]. For women less prone to FOC (low trait-anxiety), childbirth may generate a low level of negative emotional arousal and rather produce alertness and interest in the ongoing process [1]. Emotions become more intense during labour and delivery, which – in FOC women – may disrupt perception and behaviour, which in turn may lead to more uncertainty, greater concern and more intense fear [1].

### Consequences of severe FOC

Previous studies suggest that women with severe FOC and their infants are at increased risk of several adverse conditions, including hypertension and pre-eclampsia [17], pre-term birth [18, 19], complications during delivery and emergency caesarean section [20], extra use of pain medication during delivery [21, 22], prolonged delivery and trauma anxiety [23], whereas their infants may more likely suffer from low birth weight and emotional and behavioural problems [24].

### Haptotherapy

In the Netherlands HT [25] is an officially acknowledged profession. It is practiced by qualified healthcare haptotherapists who are members of and licensed by the Association of Haptotherapists [26]. Most haptotherapists have a basic education as a physical therapist. As will be explained below, healthcare haptotherapists differ from `haptonomic pregnancy counselors`, who are qualified for haptonomic counseling of healthy pregnant women not in need of therapeutic interventions [27, 28]. These pregnancy counselors are neither therapist nor do they perform any haptotherapeutic interventions. In the Netherlands, a patient with severe FOC can be referred to a specialized licensed healthcare haptotherapist. In practice some overlap exists between the domains of the healthcare haptotherapists and the haptonomic pregnancy counselors. The healthcare haptotherapists taking part in this study to accomplish the HT also perform haptonomic pregnancy counseling with pregnant women, not being part of the study. The HT method concerns both the identified FOC and at full mental state of the pregnant woman [29]. The intervention focuses on the identified anxiety issues and, subsequently, on a change in mindset which is meant to reduce FOC. HT aims to influence both the trait- and the state-component of FOC. A common component of all HT treatments is to become more familiar with perceived and experienced physical sensations [30]. It has empirically established that HT results in fear reduction as soon as a person registers feelings in his/her body (in this article meaning awareness of corporeality as the lived experience of the subject body [31]) and, more specifically for the pregnant woman, when she is able to have a perceptive participation for what is going on in her belly and pelvic area during pregnancy and childbirth. HT is claimed to facilitate the development of specific skills changing the cognitive appraisal of giving birth and labeling childbirth as a more normal and positive life event, which may ultimately lower FOC [32]. The HT sessions focus on (1) the pregnant women’s ability to open and close in reaction to the awareness of perceived impressions, (2) the affective confirmation of the mother-foetus bonding by means of the exercise in which the woman’s belly is touched by the partner and the foetus reacts, (3) skills such as the correct use of abdominal pressure during pushing at the third stage of labour, learning skills to handle painful contractions and learning to deal with labour pain in general. These skills may help to lower state-anxiety, intending women to feel more competent and more in control. In this way, the delivery might be anticipated with more trust and confidence. The partner (if present) can play an important role in the therapy and in the continuity of the skill training exercises. The practice of skill exercises at home together with a partner increases the effect of the skills. If the partner is not available, sometimes a mother, sister or (girl)friend can participate and provide social support, otherwise the pregnant woman will be guided on her own.

#### Changing the mindset

A further objective of HT is to make the individual aware of his/her capacity to allow feelings and to experience them. In other words the person learns to consciously open and close oneself for these feelings [33]. HT distinguishes between the `body-object` and `body-subject` [34]. The term `body-object` refers to the body as an object that, for example, can be examined for medical purposes. The term `body-subject` refers to the way the body is subjectively experienced [34, 35]. HT tries to make the patient aware of the difference between the body-object and the body-subject. This can be achieved by verbal explanation and experiential exercises to create physical awareness. HT in pregnancy serves the same goals as HT in general. More specifically, the pregnant woman needs to acquire the skill of opening herself for sensory impressions, exercising practical techniques for handling labour pains, easing contractions and correctly utilizing abdominal pressure for pressing. She additionally creates a mindset that helps her to better cope with the delivery process. Furthermore, HT aims to gradually shape the mindset and to teach the pregnant woman to become more (self-) confident about her ability to deliver the baby spontaneously vaginally. It is thought that increasing the woman’s self-reliance and self-confidence also results in FOC reduction.

#### Changing body-awareness and self-awareness

In women with FOC HT additionally focuses on becoming aware of or (re)discovering their own ability to experience feelings. The therapy is based on the dialogue between the haptotherapist and patient resulting in increased insight in the own capabilities of giving birth vaginally. Furthermore, skill developing exercises and direct touch by therapist and/or partner are applied, in order to promote body awareness and self-awareness. For example, a pregnant woman who undergoes a vaginal examination by a midwife or gynaecologist may feel somewhat awkward, although she might understand the necessity of such a physical examination. This is a normal reaction, because the area examined is considered as private by most women. The pregnant woman will let her body(-object) be internally examined, trying not being sensitively involved. The first author (GK) has labeled this mechanism: `*restrain internal sensitive participation*` (RISP), which can be functional to allow a stranger, such as a physician or midwife, access to one’s most private body parts. However, during childbirth it is not functional to isolate the feelings in the belly and pelvic area. A persistent RISP reaction may even form a severe obstruction, because the birth of a child requires sensitive involvement. This RISP reaction often occurs during a situation which is experienced as uncomfortable. Women with an almost permanently present RISP, lack the capacity to feel connected with their belly and pelvic area. The emotional experience of the pregnant woman with severe FOC may not be directly observable and she will not always express her fear at her own initiative. However, if a pregnant woman touches her belly in an objectifying manner and speaks about her child in an objectifying way, this may – according to clinical observations of the first author (GK) – be an indication of severe FOC. In haptotherapeutic practice it has been observed that many pregnant women with severe FOC have an undesired objectified perception of both their (lower) body and their child. HT tries to familiarize pregnant women with their body and its functions and to teach specific skills that assist in creating a positive prospect on giving birth. These skills are meant to facilitate coping with uterine contractions, to handle the labour pain more adequately and to push more effectively in the third phase of the delivery. Additionally, application of these techniques is expected to create a change in the woman’s perception of her pregnancy, which may reduce. Subsequently, a reduction of FOC may lead to fewer complications during and after birth.

### Aims

The main goal of this study is to evaluate the effectiveness of HT in reducing severe FOC, in comparison to (1) psycho education about pregnancy and childbirth, and (2) to care as usual. We also study how effectiveness is related to background variables including perceived distress, social support, and complications in pregnancy.

The following research questions are addressed:

1. Do FOC women with severe FOC after HT treatment have a lower FOC than women who receive psycho education about pregnancy and childbirth via the internet or who have care as usual?
2. Do women with severe FOC after HT treatment (1) have a better emotional bonding with their child during pregnancy and postpartum and (2) have fewer complications requiring forceps, vacuum extraction, or cesarean delivery and less third degree tears or episiotomy, (3) have lower levels of distress, depression, anxiety, PTSD symptoms and somatization, than women who receive psycho education about pregnancy and childbirth via the internet or who have care as usual?

## Methods/Design

### Study participants

The study sample consists of pregnant women, age ≥ 18, with severe FOC. FOC is measured by the Wijma Delivery Expectancy/Experience Questionnaire (W-DEQ) [15].The participants with a W-DEQ score ≥ 85 are randomly assigned to one of the three arms: (1) HT (HT Group), (2) internet psycho-education (Internet Group) or (3) care as usual (Care as usual Group). Women with a W-DEQ score < 85, and thus not classifying for any of the other three treatment arms, will be randomly assigned to (4) the Comparison-group in which one third of all participants with a W-DEQ score < 85 will be followed. Exclusion criteria are multiparity and a history of psychotic episodes.

### Ethical approval

This trial has been approved by the Dutch Medical Ethics Review Committee and registered under number NL3490000811. URL: https://www.toetsingonline.nl/to/ccmo_search.nsf/fABRpop?readform&unids=C1257BA2002CC066C1257C75003FECAC.

### Randomisation

Randomisation is arranged by computer-generated numbers by means of the program RANDOM.ORG, that has provided a random list of thousand numbers 1, 2 and 3 [36]. An eligible pregnant woman with a W-DEQ A score ≥ 85 gets a number of 1, 2 or 3 in the order of the list. Those with the numbers 1, 2, and 3 are assigned to the HT Group, the Internet Group and Care as usual Group respectively.

### Procedure

Recruitment takes place on the project’s internet website http://www.bevallingsbeleving.nl and by participating midwives, obstetricians and gynaecologists. During the routine check-up of pregnant women in gestation weeks 20–24, participating midwives and gynaecologists offer potential participants an information letter and/or a flyer that refers to the project’s internet website for registration for the study. Pregnant women show their informed consent by sending the completed approval form to the coordinating investigator, who returns the URL and login code to the participants by email. For the project a special safe internet environment has been developed, facilitating the completion of the online questionnaires. Inclusion of participants will take approximately three years, followed by one year to complete follow up measurements. The therapeutic intervention will be carried out by certified haptotherapists in various regions in the Netherlands. Admission in 20–24 weeks gestation will continue until at least 64 participants with a W-DEQ A score ≥ 85 have been included in each of the three arms respectively. At that time recruitment for the Comparison Group also stops.

### Measures

#### Background variables

General issues concerning the birth and background factors will be assessed using a questionnaire which has been especially designed for this study to collect information about demographic characteristics and the participants’ perception on health care in connection to pregnancy and delivery.

#### Fear of childbirth

The W-DEQ has been designed to measure FOC operationalised by the cognitive appraisal of the delivery. This 33-item rating scale has a 6-point Likert scale as response format, ranging from `not at all` (=0) to `extremely` (=5), yielding a score-range between 0 and 165. Internal consistency and split-half reliability of the W-DEQ = 0.87. A W-DEQ score of ≥ 85 is considered to signify severe FOC [15]. The W-DEQ proved to be a useful diagnostic test for disabling fear of childbirth in Swedish late pregnant women (sensitivity 91%, specificity 96%) [37].

#### Distress, anxiety, depression, somatization

Distress, Anxiety, Depression and Somatization will all be measured with the `Four Dimensional Symptom Questionnaire` (4DSQ) [38]. The 4DSQ consists of a list of 50 symptoms of psychological and psychosomatic symptoms according to DSM IV [4].The 4DSQ measures distress, depression, general anxiety and somatization as separate dimensions. The 4DSQ scales have a high internal consistency (Cronbach’s alpha: 0.84 to 0.94) [39, 40].

#### Social support

Social support is measured by the Social Support Questionnaire (SSQ) [41]. The SSQ is a valid instrument for measuring social support and has acceptable psychometric properties. The first part of each item assesses the number of available others the respondent feels (s)he can turn to in times of need in each of a variety of situations (Number of Perceived Availability score). The second part of each item measures the individual’s degree of satisfaction with the social support (Satisfaction score). For the subscale `number of supporters` Cronbach’s alpha is 0.90 and for the subscale `satisfaction of support` Cronbach´s alpha is 0.92.

#### Anxiety and depression

Anxiety and Depression are measured by the `Hospital Anxiety and Depression Scale (HADS) [42, 43]. The HADS includes a depression and anxiety subscale, each composed of seven items. Each item is scored on a scale ranging from 0 to 3. The HADS is widely used in medical patients, because it does not contain items relating to physical symptoms (e.g., fatigue, sleep problems) that are connected with all kind of medical and physical conditions (including pregnancy) and related to serious mental disorders. The Cronbach's alpha is: 0.91 for total scale; 0.86 for the anxiety subscale, and 0.85 for the depression subscale [42].

#### Emotional bonding

**Figure 1:**
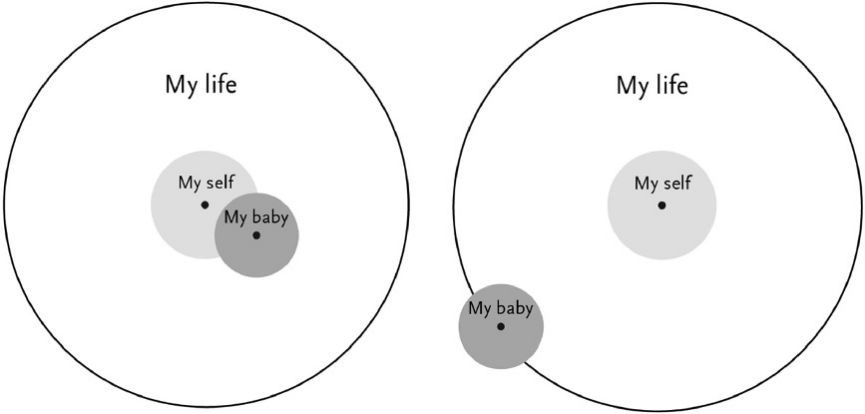
**Two examples of the pictorial representation of attachment measure.**

Emotional bonding is measured by the Pictorial Representation of Attachment Measure (PRAM) [44]. The PRAM measures mother-child bonding in a quick and easy way [44] (see Figure 1). A pregnant woman is shown a white screen with a big circle which represents her life as it currently is. A yellow circle in the centre of the big circle represents the woman's 'Self'. She is handed a green circle and is instructed to imagine that the green circle represents the unborn baby. Subsequently she was asked: "where would you place the baby in your life at this moment?" For quantitative use, the outcome measure is the Self-Baby-Distance (SBD), i.e., the distance (in centimetres) between the centres of the 'Baby' and 'Self' circles.

#### PTSD following childbirth

PTSD after childbirth is measured using the Traumatic Event Scale (TES) [23]. The TES has been developed in accordance with DSM-IV criteria for the PTSD syndrome and comprises all the DSM-IV symptoms and criteria of PTSD [4]. Internal consistency for the TES = 0.87.

#### Birth complications

Birth complications and medical interventions, such as pain relief and instrumental delivery are recorded. For this has been designed a birth evaluation questionnaire.

### Timing of measurement

**Figure 2:**
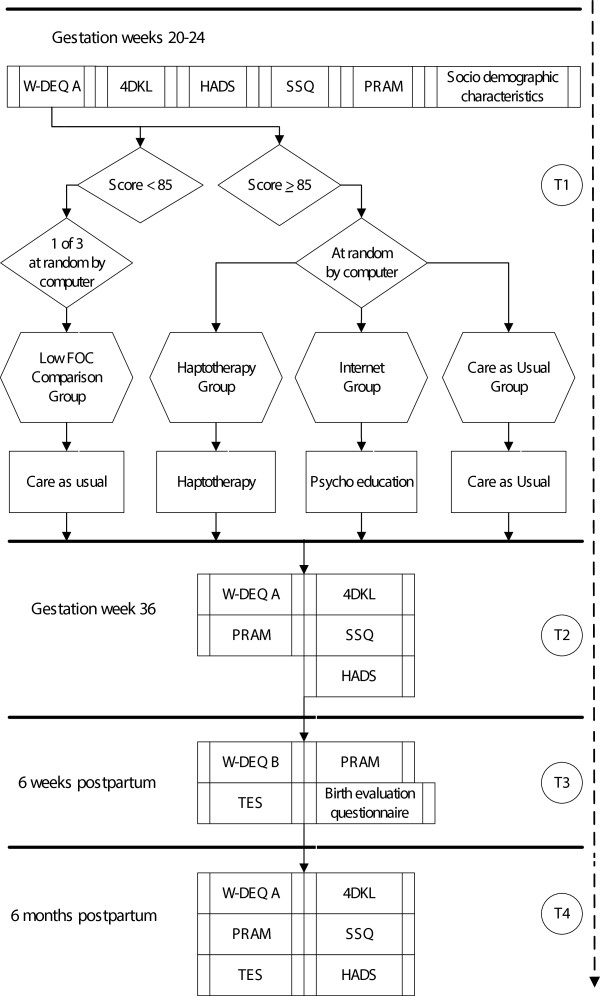
**Schematic representation of the design and procedure of this study.**

All participants answer questionnaires by internet at the following four moments (see Figure 2):

**T1:** admission to the study in weeks 20–24 of gestation. Measures: W-WDEQ A, 4DKL, HADS, SSQ, PRAM, demographic characteristics.

**T2:** week 36 of gestation. Measures: W-WDEQ A, 4DKL, HADS, SSQ, PRAM.

**T3:** 6 weeks postpartum. Measures: W-WDEQ B, PRAM, TES, Birth evaluation questionnaire.

**T4:** 6 months postpartum. Measures: W-WDEQ A, 4DKL, HADS, SSQ, PRAM, TES.

## Discussion

### The haptotherapeutic intervention

The structure of the standardized HT intervention has been developed by the first author (GK) in close collaboration with HT colleagues. The first three sessions contain simple exercises from everyday life that correspond with the pregnant woman’s own experiences, to guide her towards her own ability to open up and close herself to the awareness of feelings. For instance, by simply shaking hands differently or handling daily items differently, the woman can discover that she can choose to perform these activities either with or without the awareness of sensate focus. Next, these skills are used to distinguish between having a body as an object and the conscious intrinsic experience of the body as a subject. In subsequent sessions, these skills that the pregnant woman is developing/has developed are repeatedly reaffirmed by the haptotherapist. Next, these skills are applied to learn the correct use of abdominal pressure during pushing and handling labour pains and uterine contractions. In the practice of counseling a pregnant woman with severe FOC, it frequently appears that, instead of feeling a joyful expectancy of the foetus in her, she is extremely negatively focused on the upcoming delivery which she severely fears. In contact with the pregnant woman, the therapist speaks about the `baby or child in your womb`, instead of `foetus`. The pregnant woman may have lost her own ability to open up or shut herself in reaction to the awareness of sensational impressions. The HT exercises are designed to create a change in the woman’s perception of her pregnancy [45] and to promote a more positive attitude. Stimulating positive affective contact between both parents (if a partner is present) and the unborn child is affirmative for the woman as a mother to be. The effect is that she may feel more relaxed, more at ease, more secure and, as a consequence, the muscle tone of her uterus may decrease considerably. In addition, she may become more involved with the upcoming labour process, creating confidence which is expected to imply a decrease of her FOC. Usually it takes several weeks to help the mother develop and integrate these HT skills into daily live [46].

### The content of the sessions

HT for pregnant women with severe FOC in the present study comprises a combination of skills, taught in eight sessions of one hour between gestational week 16 and 36. Preferably, the partner of the pregnant woman also attends every session and participates actively in several exercises. If the partner, mostly the father, has the capacity for affirmative affective contact, in connection with his/her partner and their child, (s)he helps to create an atmosphere of safety, security and trust for the pregnant women. If the partner is not (yet) comfortable with this, the haptotherapist can guide him/her in this with some simple exercises. Similarly, the affective affirming contact between partners can be just self-evident. The content of the separate sessions will be briefly described below.

#### Session 1: Intake

Introduction, getting to know each other, taking an anamnesis, and making an agreement on the working methods of HT as defined in a treatment guideline. The first session is mainly an informative and administrative meeting.

#### Session 2: Ability to open and close

Introduction into the human ability to open and close to the awareness of sense impressions and the experience of its physical consequences. After this, the pregnant woman and her partner are taught to feel the difference between emotionally (affective) turning towards and turning away from another person. Once this is clear, this skill is applied in an exercise for the parents directed to the foetus by invitingly touching the woman’s belly. The foetus in the belly may respond by moving towards the touching hand. The confrontation with the ability to open up or to shut to the awareness of sensate impressions is the essence of the HT intervention which is the basis of the therapy.

#### Session 3: Further development of the ability to open and close

It is necessary to repeat the introductory exercises several times, because women with FOC, who are often blocked by their fear, may have difficulty to open up for sensate impressions. At the end of the session the exercise with invitingly touching the woman’s belly and the reactions of the baby may be further explored. The movements of the baby can help the pregnant woman to get familiar with the sensations of her body, because the movements of the baby from the inside draw the woman’s attention.

#### Session 4: Sensibilisation

HT stresses the importance of a sensitive interaction between the woman and her foetus and her (lower) body in order to facilitate the delivery proces. Whereas the first three sessions can be seen as a preparation, in the fourth session the attention will be directed to the belly and pelvic area of the pregnant woman. New exercises are performed to sensitize this part of the body. At the end of the session, the exercise in which the woman’s belly is touched and the reactions of the baby are further explored, which is meant to increase sensitivity of both parents gradually.

#### Session 5: Abdominal press

The aim of the fifth session is to practice the right use of abdominal pushing during childbirth. Therefore, all the aspects of opening for sensate impressions, interaction between the mother and her foetus, emotionally turning towards the childbirth process and emotional reactions in general will be paid attention to. At the end of the session, there again is a rehearsal of the belly touch exercise.

#### Session 6: Absorbing contractions and dealing with pain

The aim of this session is to practice coping with uterine contractions and dealing with pain during the delivery. Therefore all aspects dealt with in previous sessions, will again be paid attention to and at the end of this sixth session, as a recurrent theme, the focus again will be on invitingly touching the woman’s belly.

#### Session 7: Labour and delivery simulation

This seventh session consists of a training of labour by simulating all the aspects of labour as far as possible. Abdominal press and coping with contractions and dealing with pain in various positions are practiced. Attention is also paid to the role of the partner during labour and delivery.

#### Session 8: Evaluation and rehearsal

Evaluation of and, if necessary, rehearsal of exercises are the main components of this last session.

### Psycho education via internet condition

During gestation weeks 20–36, the Internet Group follows a course in eight modules via the Internet providing information about pregnancy and labour and delivery [47]. The entire process, from the beginning of the pregnancy to the delivery and post partum period is described. In this way, the pregnant woman can increase her knowledge about the normal course of pregnancy and delivery. Each week, the participant has the opportunity to ask questions about her own situation. The program covers a period of 8 weeks. In the first week, there is information about the development of the embryo and changes in the mother’s body in the first trimester. In the second week, the information addresses how these developments and processes continue during the second part of the pregnancy (the second trimester). The third week focuses on everything that happens in the final phase of the pregnancy (the third trimester). The fourth week begins with information on preparation for the delivery and important points to ponder in view of this major event. There is attention to what happens when labour begins. The fifth week is devoted to the options for pain management during labour and delivery, the various methods available and what the woman herself can do. In the sixth week, the course continues focussing on labour and delivery, in particular on what happens during the second and third phase of labour. In the seventh week possible emergencies that can occur during the delivery are discussed, what can happen and what is done in such case, including emergency caesarean sections. Week eight, the closing session, focus on the final part of the delivery: birth of the placenta (third stage of labour) and on the first days postpartum.

### Care as usual group

The Care as Usual Group receives care as usual according to the standards of the Royal Dutch Organisation of Midwives (Koninklijke Nederlandse Organisatie van Verloskundigen, KNOV) and the Dutch Organization of Obstetrics and Gynaecology (Nederlandse Vereniging voor Obstetrie en Gynaecologie, NVOG).

### Low FOC comparison group

The low FOC Comparison Group receives care as usual.

## Statistical analyses and power analysis

### Statistical analyses

To answer the primary research questions, we will use descriptive statistics (M and SD), between-subjects analysis of variance (ANOVA), followed by two planned pair wise comparisons (HT treatment versus care as usual, and HT treatment versus internetgroup).To maintain the experiment-wise Type I error rate at the 5% level, we use a Bonferroni corrected alpha of 0.05/2 = 0.025 for each single comparison. Clinical significance of the HT intervention will be tested both according to Jacobson and Truax’s criteria of reliable and clinical change [48] and by examination of the number of participants changing W-DEQ score from above to below a cut off score of 85. For the secondary research questions, we will use multiple regression, with `distress, depression, anxiety, somatization, PTSD symptoms, birth complications` as dependent variables, intervention (care as usual, psycho education via internet, and haptotherapy) as independent variable, and `background variables, social support, FOC` as covariates.

### Sample size calculation

The power analysis concerns the primary research question. All computations are performed using GPower3.0 [49]. For the planned pair wise comparisons, to detect medium effects or larger (i.e., Cohen’s *d* ≥ 0.5; [50]) with at least 80% power and a Bonferroni corrected alpha of 0.025, a minimal sample size of 64 in each group is needed. With three groups (one experimental and two control groups) and 64 respondents per group, we also have at least 80% power to find effect sizes (*n*^*2*^) in excess of .05 (indicating a medium effect according to Cohen [50] using ANOVA.

## Abbreviations

ANOVA: Analysis of variance
DSM-IV: Diagnostic and statistical manual of mental disorders IV
FOC: Fear of childbirth (as measured by the W-DEQ)
PRAM: Pictorial representation of attachment measure
Severe FOC: W-DEQ score ≥ 85
State-Anxiety related to childbirth: Situational fear of childbirth
Trait-Anxiety related to childbirth: Disposition to react with fear of childbirth
TES: Traumatic event scale
W-DEQ: Wijma delivery expectance/experience questionnaire
W-DEQ A: W-DEQ, Version A measures prepartum fear of delivery
W-DEQ B: W-DEQ, Version B measures postpartum fear of delivery
4DSQ: Four dimensional symptom questionnaire.
